# Viral and atypical bacterial aetiologies of infection in hospitalised patients admitted with clinical suspicion of influenza in Thailand, Vietnam and Indonesia

**DOI:** 10.1111/irv.12326

**Published:** 2015-10-13

**Authors:** Heiman F L Wertheim, Behzad Nadjm, Sherine Thomas, Suhud Malik, Diep Ngoc Thi Nguyen, Dung Viet Tien Vu, Kinh Van Nguyen, Chau Vinh Van Nguyen, Liem Thanh Nguyen, Sinh Thi Tran, Thuy Bich Thi Phung, Trung Vu Nguyen, Tran Tinh Hien, Uyen Hanh Nguyen, Walter Taylor, Khanh Huu Truong, Tuan Manh Ha, Kulkanya Chokephaibulkit, Jeremy Farrar, Marcel Wolbers, Menno D de Jong, H Rogier van Doorn, Pilaipan Puthavathana

**Affiliations:** a Wellcome Trust Major Overseas Program, Oxford University Clinical Research UnitHanoi, Ho Chi Minh City, Vietnam; b Nuffield Department of Clinical Medicine, Centre for Tropical Medicine, University of OxfordOxford, UK; c National Institute of Health Research and DevelopmentJakarta, Indonesia; d National Hospital of Tropical DiseasesHanoi, Vietnam; e Hospital for Tropical DiseasesHo Chi Minh City, Vietnam; f National Hospital of PediatricsHanoi, Vietnam; g Mahidol Oxford University Clinical Research UnitBangkok, Thailand; h Children's Hospital No 1Ho Chi Minh City, Vietnam; i Children's Hospital No 2Ho Chi Minh City, Vietnam; j Siriraj HospitalBangkok, Thailand; k Academic Medical Center, University of AmsterdamAmsterdam, The Netherlands

**Keywords:** Hospitalisation, influenza, *Mycoplasma pneumoniae*, respiratory tract infections, rhinovirus, RSV

## Abstract

**Background:**

Influenza constitutes a leading cause of morbidity and mortality worldwide. There is limited information about the aetiology of infection presenting clinically as influenza in hospitalised adults and children in South-East Asia. Such data are important for future management of respiratory infections.

**Objectives:**

To describe the aetiology of infection presenting clinically as influenza in those hospitalised in South-East Asia.

**Methods:**

Respiratory specimens archived from July 2008 to June 2009 from patients hospitalised with suspected influenza from Indonesia, Thailand and Vietnam were tested for respiratory viruses and atypical bacteria by polymerase chain reaction.

**Results:**

A total of 1222 patients’ samples were tested. Of 1222, 776 patients (63·5%) were under the age of 5. Viruses detected included rhinoviruses in 229 of 1222 patients (18·7%), bocaviruses in 200 (16·4%), respiratory syncytial viruses in 144 (11·8%), parainfluenza viruses in 140 (11·5%; PIV1: 32; PIV2: 12; PIV3: 71; PIV4: 25), adenovirus in 102 (8·4%), influenza viruses in 93 (7·6%; influenza A: 77; influenza B: 16) and coronaviruses in 23 (1·8%; OC43: 14; E229: 9). Bacterial pathogens were *Mycoplasma pneumoniae* (*n* = 33, 2·7%), *Chlamydophila psittaci* (*n* = 2), *C. pneumoniae* (*n* = 1), *Bordetella pertussis* (*n* = 1) and *Legionella pneumophila* (*n* = 2). Overall, in-hospital case fatality rate was 29 of 1222 (2·4%).

**Conclusion:**

Respiratory viruses were the most commonly detected pathogens in patients hospitalised with a clinical suspicion of influenza. Rhinovirus was the most frequently detected virus, and *M. pneumoniae,* the most common atypical bacterium. The low number of detected influenza viruses demonstrates a low benefit for empirical oseltamivir therapy, unless during an influenza outbreak.

## Introduction

Influenza is a common reason for primary care consultation and constitutes a leading cause of hospitalisation, morbidity and mortality worldwide.^1^ Non-influenza viruses are the most common causes of illness that clinically resemble influenza, particularly in children. Data on the epidemiology and disease burden of influenza-related disease in South-East Asia (SEA) are emerging, and this, in turn, is shedding more light on the epidemiology of other viral and bacterial aetiologies of influenza-like illnesses (ILIs) in hospitalised adults and children in this region.^2^ The insight provided by this information is important not only for future prevention strategies, treatment and clinical management of respiratory infections, but also to guide future studies in this region.

Recently, many diagnostic advances have been made in order to help to determine the aetiology of acute respiratory infections. These advances have been made possible by molecular diagnostic techniques that are now in widespread use. Furthermore, research in this field has gained much attention and funding due to the outbreaks of severe acute respiratory syndrome coronavirus (SARS-CoV) and avian influenza viruses A/H5N1 and A/H7N9 in SEA and Middle Eastern respiratory syndrome coronavirus (MERS-CoV) in the Middle East.

Generally, the causative agents of ILI remain undiagnosed in routine clinical practice due to the expense of testing and the slow turnaround time for most diagnostic polymerase chain reactions (PCRs), considered too long to impact the management of usually self-limiting respiratory tract infections. However, with the increasing use of antiviral drugs, such as neuraminidase inhibitors for influenza, testing for ILI pathogens may become more important for rational antiviral drug treatment and to prevent overuse of antibiotics, thereby helping to control costs and prevent the emergence and spread of antimicrobial drug resistance.

During the past few years, several novel causative agents of ILI have been identified in respiratory specimens, including human metapneumovirus, new human coronaviruses (NL63, HKU1, SARS-CoV, MERS-CoV), rhinovirus C and continuously emerging novel lineages and subtypes of influenza virus A capable of infecting humans (H5N1, H5N6, H1N1pdm09, H3N2v, H7N7, H7N9, H9N2, H6N1, H10N8). Viruses for which disease causation has not (yet) been established have also been identified, including human bocavirus and respiratory polyomaviruses, and likely there are many more to come.^3^–^6^ The most comprehensive data on the causes of ILI originate from developed countries, with some data available from SEA. Recently, studies have been published from Asian countries,^7^–^12^ providing much needed, valuable information and showing a similar spectrum of viral pathogens.

This study was designed to determine the viral and atypical bacterial aetiologies of ILIs in patients in SEA using molecular techniques. The detection of influenza viruses and respiratory pathogens other than influenza viruses in specimens that were sent for routine influenza testing can provide valuable insights into the epidemiology of ILI across all age groups in three SEA countries over the same time period. Furthermore, this study could address the potential occurrence of multiple simultaneous infections in the same patient.

## Methods

We performed a laboratory-based surveillance study, in which we tested all archived respiratory specimens from hospitalised patients over 1 year (July 2008 to June 2009) with clinically diagnosed ILI meeting the following criteria: age ≥1 year, signs of ILI according to treating physician, duration of ILI symptoms ≤10 days and respiratory specimens sent for influenza testing. Clinicians at all participating hospitals received regular training in WHO protocols for the management and recognition of influenza/ILI. Archived respiratory specimens (nose swab, throat swab, nasopharyngeal aspirate, nasal wash, tracheal aspirate, bronchoalveolar lavage) were obtained from 12 sites in three countries across SEA (three sites in Indonesia, four sites in Thailand, five sites in Vietnam). Influenza testing capacity was created for a randomised clinical study comparing two dosages of oseltamivir for severe influenza by sites in the South East Asia Infectious Diseases Clinical Research Network, as described elsewhere.^13^ The specimens used for this study were stored at −80°C until further testing. Where more than one specimen type was available for each patient, they were prioritised in the sequence endotracheal aspirate/bronchoalveolar lavage > nasopharyngeal aspirate > throat swab > nasal swab/washing. This retrospective study for additional testing on archived specimens after full anonymisation of specimens was approved by the institutional review boards of each site.

### Laboratory testing

Commercially available multiplex polymerase chain reaction (PCR)/gel electrophoresis assays (Seeplex® RV12 ACE Detection and Seeplex® PneumoBacter ACE; Seegene, South Korea^14^–^16^) were used to detect 12 major respiratory viruses and six respiratory bacterial pathogens: influenza viruses A and B, respiratory syncytial viruses (RSVs) A and B, rhinoviruses A/B, coronaviruses OC43/HKU1 and 229E/NL63, adenovirus, parainfluenza viruses 1–3, human metapneumovirus, *Mycoplasma pneumoniae*, *Chlamydophila pneumoniae, Legionella pneumophila* serotype 1 and *Bordetella pertussis*. Additional in-house real-time [reverse-transcriptase (RT)] PCRs were used to detect the following viruses: rhinoviruses A, B and C, enteroviruses, parainfluenza virus 4, bocavirus, parechoviruses and C*hlamydophila psittaci*, as described elsewhere.^17^–^19^ Basic demographic data (country, sex, age) were collected as well as date of disease onset, date of admission, type of specimen and intensive care unit (ICU) admission. For the analysis results of *Streptococcus pneumoniae* and *Haemophilus influenzae*, PCRs were not taken into account, because of the high positivity rates resulting from nasopharyngeal carriage of 25·4% and 31·4%, respectively, in this study (data not shown).

## Analysis

The baseline demographic characteristics (age category, sex, country of admission and proportion of ICU admissions) were summarised as frequency and proportion. The frequencies of different pathogens were summarised per country, sex and age category to determine the most frequent viruses and bacteria in each stratum.

Disease outcomes including severity (severe defined as ICU admission), death, duration of hospitalisation and duration of illness were examined by regression analysis. The dependence of binary outcomes (severe disease and death) on the presence of pathogens was analysed by logistic regression, adjusted for age and sex.

Kaplan–Meier survival analysis was used to visualise the number of hospitalisation days between each age category (patient deaths were censored). Cox regression was used to analyse whether viruses were found more often if the duration of illness before presentation to hospital was shorter. Regression analysis was based on the data from Vietnam and Thailand only as relevant clinical data were missing from Indonesia.

The statistical software sas version 9.2 (SAS Institute Inc., Cary, NC, USA) was used for all computations. A two-sided *P* value of 0·05 or less was interpreted as statistically significant.

## Results

### General

A total of 1222 patient samples were collected at participating sites. Table1 summarises the demographic data. The median number of days from illness onset to specimen collection was 4 (IQR: 3–6 days). For 155 patients, no exact admission date was known. The majority of patients in this study were under the age of 5 (776, 63·5%). Most patients were enrolled in Vietnam (*n* = 826, 67·6%). The median age was higher in Indonesian patients as compared to those from Vietnam and Thailand (16 year, versus 4 year and 2 year, respectively). One hundred and 67 patients (13·7%) were admitted to the intensive care unit (ICU); ICU admission data from 232 patients were unknown (Indonesia: 223; Thailand: 2; Vietnam: 7). Sixty-seven patients (5·5%) were mechanically ventilated and the overall reported in-hospital mortality was 2·4% (*n* = 29).

**Table 1:** Basic demographic data of patients sampled

	No. of patients		Total cases (*n* = 1222)
	Male (%)	Female (%)	*N* (%)
Age range in years			
<5	452 (58·2)	324 (41·8)	776 (63·5)
5–14	73 (57·9)	53 (42·1)	126 (10·3)
15–44	98 (50·0)	98 (50·0)	196 (16·0)
45–64	52 (67·5)	25 (32·5)	77 (6·3)
>65	26 (55·3)	21 (44·7)	47 (3·9)
Total	701 (57·4)	521 (42·6)	1222
Country			
Vietnam	500 (60·5)	326 (39·5)	826 (67·6)
Indonesia	114 (50·7)	111 (49·3)	225 (18·4)
Thailand	87 (50·9)	84 (49·1)	171 (14·0)
Intensive care admission			
Vietnam	88 (62·9)	52 (37·1)	140/826 (17·0)
Indonesia	na	na	na
Thailand	10 (40·0)	15 (60·0)	25/171 (14·6)

na, not available.

### Aetiology

**Figure 1:**
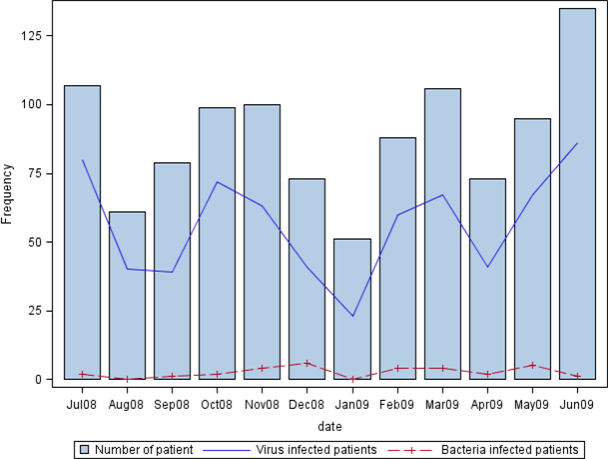
Number of viral or atypical bacterial pathogens detected by month (note: those with no exact admission date were excluded).

Respiratory pathogens were detected in 741 of 1222 patients (60·6%): viruses in 716 (58·6%), atypical bacteria in 39 (3·2%) and both in 14 (1·2%; Figure1). No clear seasonality of the number of patients enrolled per month was seen during the year that the samples were collected.

The viruses detected were rhinoviruses in 229 of 1222 patients (18·7%), bocaviruses in 200 (16·4%), RSV in 144 (11·8%), parainfluenza viruses in 140 (11·5%; PIV1: 32; PIV2: 12; PIV3: 71; PIV4: 25), adenovirus in 102 (8·4%), influenza viruses in 93 (7·6%; influenza A: 77; influenza B: 16), human metapneumoviruses in 22 (1·8%), coronaviruses in 23 (1·8%; OC43: 9; E229: 14), enteroviruses in 53 (4·3%) and parechoviruses in 5 (0·4%).

**Figure 2:**
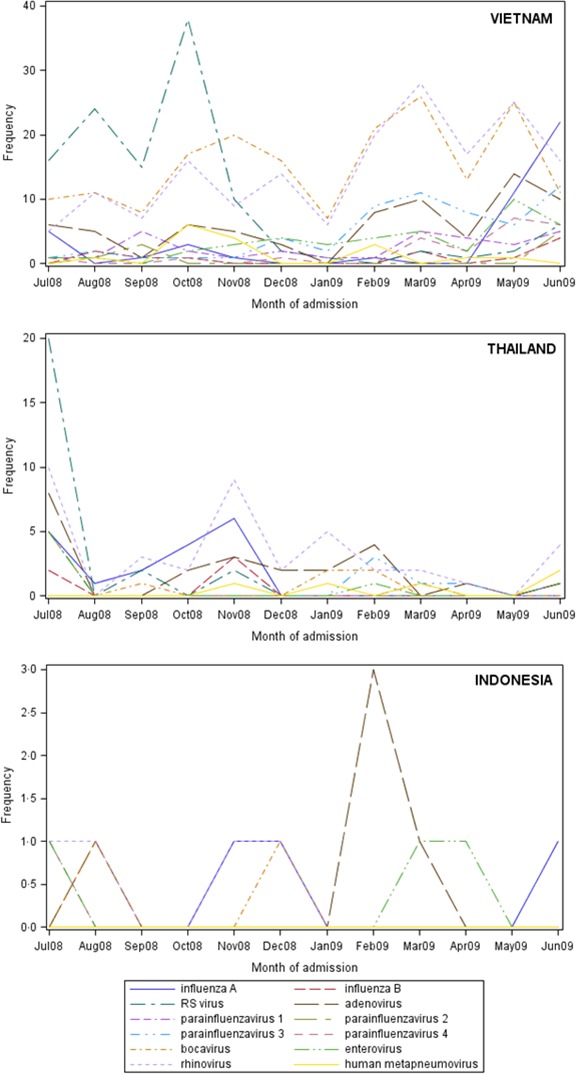
Frequency of detected viruses by country.

Detected bacterial pathogens were *M. pneumoniae* (*n* = 33, 2·7%), *C. psittaci* (*n* = 2), *C. pneumoniae* (*n* = 1), *B. pertussis* (*n* = 1) and *L. pneumophila* (*n* = 2). Fluctuations in frequencies were found in particular months for several viruses, with RSV occurring from July to November 2008 and rhinovirus peaking in March 2009 (Figure2).

Due to differences in age group representation, the number of viral agents detected per patient was lower in Indonesia (60/225, 0·27) than in Vietnam (800/826, 0·97) and Thailand (151/171, 0·88). Likewise, the proportion of identified viral agents detected that were RSV was higher in Thailand and Vietnam than in Indonesia (24/151, 15·9%; 117/800, 14·6%, respectively, compared with 3/60, 5%) whilst coronavirus was a greater proportion of identified viral agents in Indonesia (7/60, 11·7%) than Thailand (3/151, 2·0%) and Vietnam (11/800, 1·4%). See Table S1 for pathogens detected by country.

There were 234 of 1222 patients (19·2%) who had at least two pathogens detected (bacterial and/or viral). Of these, 170 patients were found to have two pathogens, 54 patients had three pathogens, nine patients had four pathogens, and one patient had five pathogens. The frequency of each pathogen is shown in Table S2. The pathogens most commonly detected in patients with two or more pathogens were bocavirus (*n* = 132) and rhinovirus (*n* = 123). The proportion of patients who had either a virus or a bacterium detected appeared to decrease with age, with the highest proportion of pathogens detected in the 0–4 age group, but increased again in those older than 65. Most viral pathogens were more common in children, with parechovirus and parainfluenza virus 4 only found in children (Table2). *M. pneumoniae* was found only in patients younger than 45 years, whilst both cases of *C. psittacii* occurred in patients 45 years and above.

**Table 2:** Viral pathogens in ILI patients by age category

Age group (year)	Number of agents found (% of tested)															No. positive/No. tested
	Infl A	Infl B	RSV	OC43	E229	Adeno virus	Parainfl 1	Parainfl 2	Parainfl 3	Parainfl 4	Bocavirus	Enterovirus	Parechovirus	Rhinovirus	hMPV	
0–4	24 (3·1)	4 (0·5)	136 (17·5)	5 (0·6)	8 (1)	88 (11·3)	25 (3·2)	5 (0·6)	60 (7·7)	24 (3·1)	190 (24·5)	45 (5·8)	5 (0·6)	188 (24·2)	20 (2·6)	827/776*
05–14	17 (13·5)	3 (2·4)	3 (2·4)	0 (0)	2 (1·6)	11 (8·7)	0	1 (0·8)	1 (0·8)	1 (0·8)	8 (6·3)	5 (4)	0	21 (16·7)	1 (0·8)	74/126
15–44	24 (12·2)	4 (2)	3 (1·5)	1 (0·5)	4 (2)	3 (1·5)	6 (3·1)	6 (3·1)	10 (5·1)	0	1 (0·5)	2 (1)	0	13 (6·6)	0	77/196
45–64	7 (9·1)	1 (1·3)	1 (1·3)	1 (1·3)	0	0	1 (1·3)	0	0	0	1 (1·3)	0	0	3 (3·9)	1 (1·3)	16/77
≥65	5 (10·60)	4 (8·5)	1 (2·1)	2 (4·3)	0	0	0	0	0	0	0	1 (2·1)	0	4 (8·5)	0	17/47
Total	77 (6·3)	16 (1·3)	144 (11·8)	9 (0·7)	14 (1·1)	102 (8·3)	32 (2·6)	12 (1)	71 (5·8)	25 (2)	200 (16·4)	53 (4·3)	5 (0·4)	229 (18·7)	22 (1·8)	1011/1222

Infl A, influenza A; Infl B, influenza B; RSV, respiratory syncytial virus; oc43, coronavirus OC43; e229, coronavirus e229; Parainfl, parainfluenza virus; hMPV, human metapneumovirus.

* More positive results than samples tested due to samples with multiple positives.

The ICU admissions for each country are shown in Table1. The number of patients who were mechanically ventilated was 42 of 826 (5·1%) in Vietnam and 21 of 171 (12·3%) in Thailand (data not available for Indonesia). For the Vietnamese data set, where data were most complete, ICU admission was related to age; 15 of 616 children (2·4%) aged under 5 years, three of 36 (8·3%) aged 5–14 years, 69 of 106 (65·1%) aged 15–44 years, 33 of 45 (73·3%) aged 45–64 years and 20 of 23 (87%) aged over 65 years were admitted to ICU (χ^2^ for trend *P* < 0·0005). Logistic regression was performed on this data set including the covariates: pathogen type (virus, bacterial, both and neither), age and sex. The analysis showed that only higher age was associated with ICU admission (adjusted OR for ICU admission in those aged >64 years compared to those aged <5 years: 243·79, *P* < 0·001, Table3). No specific pathogen was associated with ICU admission.

**Table 3:** Factors associated with intensive care unit (ICU) admission in patients from sites in Vietnam and Thailand

	Total number	Number admitted to ICU	Odds ratio	Adjusted OR	95% CI (AOR)	*P* (AOR)
Viral pathogen detected	527	55	1	1	–	–
Bacterial pathogen detected	19	5	3·07	1·82	0·351–9·414	0·477
Both pathogens detected	12	0	N/A	N/A	N/A	N/A
Neither pathogen detected	261	80	3·79	1·30	0·714–2·374	0·390
Age: 0–4 years	616	15	1	1	–	–
Age: 5–14 years	36	3	3·64	3·46	0·93–12·88	0·064
Age: 15–44 years	101	69	86·39	79·78	40·74–156·23	<0·0001
Age: 45–64 years	43	33	132·22	114·07	45·02–289·02	<0·0001
Age: 65+ years	23	20	266·94	243·79	64·58–920·26	<0·0001
Male sex	495	88	1	1	–	–
Female sex	324	52	0·88	1·07	0·60–1·91	0·830

There were only sufficient data available to analyse the duration of hospitalisation for patients in Vietnam. The median duration of hospitalisation was 5 days (IQR: 3–7 days). Multivariate Cox regression analysis for hospitalisation days by pathogen type (virus, bacterial, both and neither), sex and age was performed, and both pathogen type (*P* = 0·0004) and age category (*P* = 0·015) had a significant effect. It was found that patients in the >65-year group were hospitalised longer, but did not differ by sex. Influenza A virus-positive patients were admitted for a median of 4 days (IQR: 3–8 days) versus 5 days (IQR: 3–8 days) for patients where influenza A was not detected. A similar pattern was seen for coronavirus e229: median of 3 days (IQR: 2–4 days) for positive patients versus 5 days (IQR: 3–8 days) in the negative group. Patients who were *M. pneumoniae*-positive were admitted longer: a median of 9 days (IQR: 7–12 days) versus a median of 5 days (IQR: 3–8 days). There were 29 deaths and a viral pathogen was detected in 12 (41%) of these: influenza virus A (*n* = 3), rhinovirus (*n* = 3), bocavirus (*n* = 1), coronavirus (*n* = 2), parainfluenza virus (*n* = 1), RSV (*n* = 1) and one combination of influenza A with adenovirus and rhinovirus. Data concerning the cause of death were not collected in this study; consequently, conclusions about causation cannot be drawn.

## Discussion

Influenza-like illnesses are a major cause of mortality and morbidity worldwide, especially in younger and elderly age groups. It has been suggested that a large proportion of ILI-attributable deaths globally occur in Africa and SEA.^20^ Data regarding the aetiology of respiratory infections in SEA are limited, with some of the available data suggesting that viruses account for a large proportion of these infections.^21^

In this 1-year study, we used molecular techniques to detect viruses and atypical bacteria from samples collected from patients hospitalised with ILI. The majority of patients enrolled in this study were enrolled in Vietnam and were under the age of 5. For ILI diagnostics and surveillance, nose and throat swabs are considered the sampling methods of choice, whereas for the diagnostics of *H. influenzae* and *S. pneumoniae,* Gram-staining and bacterial culture of representative purulent sputum, blood culture or urinary antigen tests (for *S. pneumoniae*) would be the testing method of choice, which were not done. Detection rates of these two pathogens were therefore not included in the analysis.

In this study, a potential pathogen was identified in 60·6% of patients. This rate is higher than the previously published rates of between 35% and 47% of organisms identified, in studies looking at the aetiology of respiratory tract infections in different Asian countries.^9^,^22^ A total of 58·6% of the study cohort were found to be positive for viruses compared to just 3·2% of the cohort who had an atypical bacterial pathogen alone detected. More recent studies form Asian countries using molecular techniques have shown the rates of virus detection reaching 72%.^10^ The most common viruses detected in our study were rhinoviruses, accounting for 32% of the patients in whom viral pathogens were detected and 18·7% of the cohort as a whole, similar to other studies.^1^,^23^ Variation between the three countries in the proportion of patients in whom viral pathogens were detected, and variations in the relative importance of different viruses, largely represents the differing age groups sampled, with the younger patients in Vietnam and Thailand having higher a proportion of RSV detected.

Bocaviruses were first described in 2005, and since then, numerous studies have described their detection in the human respiratory tract^3^ and their possible association with disease, but causation has not convincingly been proven.^24^ In this study, this virus was detected in 16·4% of patients, which is similar to other studies where detection rates ranged from 1·5% to 21·5%.^25^,^26^ These previous studies as well as this study have detected bocavirus, predominantly from young children rather than from adults. In this study as in other studies, bocaviruses were commonly (136/200, 68% bocavirus isolates in our study, Table S2) detected in combination with other pathogens, most commonly rhinovirus.

Atypical bacteria were rarely (3·2%) detected. This rate of detection is lower than previously published data, where detection varied from 13% to 26%.^27^,^28^ However, as we did not include serological diagnostic methods for atypical bacteria, the true prevalence in our patients may have been higher. The most commonly detected atypical bacteria in this study were *M. pneumoniae*, followed by *C. psittaci* and *L. pneumophila*. The results from our study are similar to the aetiological studies of community-acquired pneumonia in Singapore and Malaysia, in which *M. pneumoniae* was the commonest atypical pathogen detected.^29^ Very little is known about the epidemiology of *C. psittaci* in SEA, and indeed this pathogen has only very recently been described as causing human disease in Vietnam.^30^

The type of pathogen detected was significantly associated with the duration of hospitalisation. Atypical bacteria and in particular infection with *M. pneumoniae* was associated with longer duration of hospitalisation. The age of the patients was also significantly associated with longer duration of hospitalisation, with the >65 years age group requiring longer admissions. This probably corresponds with increasing co-morbidities and decreasing immunity with age, similar to other regions in the world. No clear seasonality was seen with the various pathogens detected, but rates of infection appeared to be higher in the winter and spring months and a study over a single year may miss seasonal trends. Rates of influenza virus A increased in Indonesia and Vietnam in April 2009 coinciding with an epidemic of seasonal H3N2 influenza in Vietnam, prior to the first sporadic cases of H1N1pdm09 detected at the end of May 2009.^31^

The strengths of this type of study include the large numbers of patients who were enrolled prospectively from three different countries in South-East Asia allowing an overview of the viral and atypical bacterial causes of ILIs in the region. This study also used RT-PCR techniques; however, an important limitation was the lack of microscopy and sputum cultures for common bacteria associated with ILIs such as *S. pneumoniae* and *H. influenzae* type b and serology for atypical pathogens. Also, limited clinical data were recorded for these cases, making it difficult to assess the clinical significance of the various pathogens detected and the virulence of each pathogen. Although swabs from all patients meeting our eligibility criteria were analysed, we have no data concerning the number of patients who may have been admitted with ILI but not swabbed. The lack of clinical data also limits comparison between sites as differing admission criteria may impact on the distribution of pathogens. Similarly, both the lack of clinical data and the absence of data concerning overall admission limit our ability to interpret the findings relating to ICU admission; the finding that adult patients were overrepresented amongst those admitted to ICU could represent differential routine sampling of adults and children on such units; however, we feel this is unlikely. There was a lack of data related to patients and co-morbidities, such as immunosuppression and COPD, which could also have had an impact in the severity of infection seen. Furthermore, a lack of suitable control patients also limits our ability to determine the attributable fraction of ILI that is likely to be caused by each virus and also prevents useful analysis of the relevance of the high rates of nasopharyngeal carriage of *S. pneumoniae* and *H. influenzae* type b.

Establishing the aetiology of ILI is becoming increasingly important, not only to establish the burden of various pathogens in order to improve management and prevention, but also to guide antiviral treatment and to try to limit the inappropriate use of antimicrobials to reduce the emergence and spread of antibiotic resistance. Our study, along with overwhelming evidence from many other studies from these and other countries, suggests that viral causes of ILI are much more common than bacterial causes, but that the viral aetiology may vary in space and time. In recent years, the emergence of SARS-CoV, MERS-CoV and avian influenza viruses A/H5N1 and A/H7N9 has highlighted the risks posed by respiratory viral infections in humans and SEA has become an area of increasing interest for monitoring pathogens. Very little has been published from this region regarding the incidence and prevalence of viral or atypical organisms that can contribute to ILIs, making studies looking at this increasingly important.

## Supporting Information

Table S1 Frequency of detected pathogens by country. **Table S2.** Frequency of pathogens found in a combination with another pathogen.
