# Supplementary material for: Viral and atypical bacterial aetiologies of infection in hospitalised patients admitted with clinical suspicion of influenza in Thailand, Vietnam and Indonesia
Source: Influenza Other Respir Viruses. 2015 Oct 13;9(6):315–22. doi: 10.1111/irv.12326 (PMC4605413; doi:10.1111/irv.12326)
Supplement: Table S1 — Frequency of detected pathogens by country. Table S2. Frequency of pathogens found in a combination with another pathogen. [file irv0009-0315-sd1.docx]

**Table S1** Frequency of detected pathogens by country.

| **Pathogen** | **INDONESIA**  **N = 225** | **THAILAND**  **N = 171** | **VIETNAM**  **N = 826** | **Total** |
| --- | --- | --- | --- | --- |
| **Influenza virus A** | 14 6.2% | 19 11.1% | 44 5.3% | 77 |
| **Influenza virus B** | 0 0% | 5 2.9% | 11 1.3% | 16 |
| **Respiratory Syncytial virus** | 3 1.3% | 24 14.0% | 117 14.2% | 144 |
| **Coronavirus OC43** | 2 0.9% | 2 1.2% | 5 0.6% | 9 |
| **Coronavirus E229** | 5 2.2% | 1 0.6% | 8 1.0% | 14 |
| **Adenovirus** | 6 2.7% | 24 14.0% | 72 8.7% | 102 |
| **Parainfluenza virus 1** | 1 0.4% | 0 0% | 31 3.8% | 32 |
| **Parainfluenza virus 2** | 2 0.9% | 1 0.6% | 9 1.1% | 12 |
| **Parainfluenza virus 3** | 3 1.3% | 10 5.9% | 58 7.0% | 71 |
| **Parainfluenza virus 4** | 1 0.4% | 2 1.2% | 22 2.7% | 25 |
| **Bocavirus** | 5 2.2% | 10 5.9% | 185 22.4% | 200 |
| **Enterovirus** | 5 2.2% | 7 4.1% | 41 4.9% | 53 |
| **Parechovirus** | 0 0% | 1 0.6% | 4 0.5% | 5 |
| **Rhinovirus** | 12 5.3% | 40 23.4% | 177 21.4% | 229 |
| **Human Metapneumovirus** | 1 0.4% | 5 2.9% | 16 1.9% | 22 |
| **Total viral agents** | **60** | **151** | **800** | **1011** |
| ***Mycoplasma pneumoniae*** | 0 0% | 6 3.5% | 27 3.3% | 33 |
| ***Chlamydophila pneumoniae*** | 0 0% | 1 0.6% | 0 0% | 1 |
| ***Chlamydophila psittacii*** | 0 0% | 0 0% | 2 0.2% | 2 |
| ***Legionella pneumophila*** | 0 0% | 0 0% | 2 0.2% | 2 |
| ***Bordetella pertussis*** | 1 0.4% | 0 0% | 0 0% | 1 |
| **Total atypical bacteria** | **1** | **7** | **31** | **39** |

**Table S2**. Frequency of pathogens found in a combination with another pathogen.

| **Pathogen** | **Number of agents found in combination with another pathogen (%)** |
| --- | --- |
| Bocavirus | 136/200 (68) |
| Rhinovirus | 127/229 (55.5) |
| Adenovirus | 66/102 (64.7) |
| Respiratory Syncytial virus | 54/144 (37.5) |
| Enterovirus | 45/53 (84.9) |
| Parainfluenza virus 3 | 41/71 (57.7) |
| Influenza virus A | 18/77 (23.4) |
| *Mycoplasma pneumoniae* | 13/33 (39.4) |
| Parainfluenza virus 1 | 12/32 (37.5) |
| Parainfluenza virus 4 | 11/25 (44) |
| Parainfluenza virus 2 | 7/12 (58.3) |
| Coronavirus OC43 | 6/9 (66.7) |
| Coronavirus E229 | 6/14 (64.3) |
| Influenza virus B | 5/16 (31.3) |
| Parechovirus | 5/5 (100) |
| Human metapneumovirus | 5/22 (22.7) |
| *Bordetella pertussis* | 1/1 (100) |
| Number of patients infected with 2 agents = 170 (13.9%)  Number of patients infected with 3 agents = 54 (4.4%)  Number of patients infected with 4 agents = 9 (0.7%)  Number of patients infected with 5 agents = 1 (0.1%) | |
